# A Curious Case of Cutaneous Horn

**DOI:** 10.7759/cureus.10253

**Published:** 2020-09-05

**Authors:** Gorrepati Rohith, Souradeep Dutta, Sreenath G. S.

**Affiliations:** 1 General Surgery, Jawaharlal Institute of Postgraduate Medical Education and Research, Puducherry, IND; 2 Surgery, Jawaharlal Institute of Postgraduate Medical Education and Research, Puducherry, IND

**Keywords:** cutaneous horn

## Abstract

Cutaneous horns are uncommon lesions that occur as conical projections in various sun-exposed parts of the body. They are made of hyperkeratotic material and look similar to animal horns. These lesions lack a central bony core that differentiates them from the animal horns. They are frequently associated with various benign, premalignant, and malignant lesions, and therefore carry a clinical significance. We present a case of a 58-year-old gentleman who presented with a conical projection over the center of the forehead, which was clinically diagnosed as a cutaneous horn. Excision of the horn along with the base was done, and the histopathology revealed benign seborrheic keratosis as the underlying lesion. The excision of small lesions along with their bases or biopsy of the base of large lesions is of paramount importance in the management of cutaneous horns.

## Introduction

Cutaneous horns (cornu cutaneum/devil’s horn) are unusual epidermal hyperkeratotic lesions resembling an animal horn [1]. Most of these lesions occur in varied shapes either curved or straight. Their size may range from few millimeters to several centimeters. They mostly occur in sun-exposed parts, such as the face, forearms, and dorsum of hands [2]. There appears to be no sex predilection, but the chances of presence of malignancy at the lesion’s base are more in males compared to females [3]. Around 60% of these lesions are benign, whereas the rest of the lesions may harbor a variety of premalignant or malignant conditions at their base [4]. Owing to this, shaving the lesion off its base is discouraged and complete excision of the lesion along with the base followed by complete histopathological examination is to be carried out to identify the underlying pathology [5].

## Case presentation

**Figure 1:**
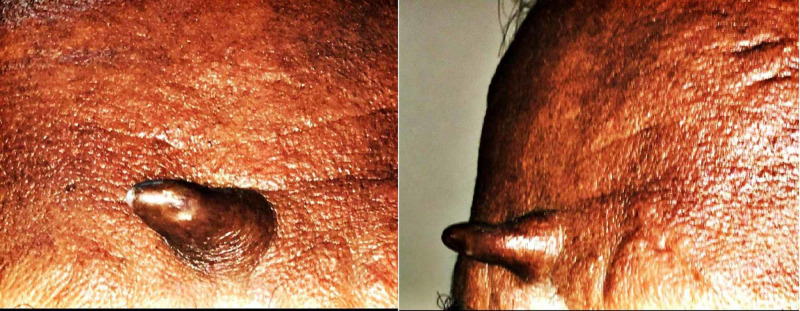
A 1.5-cm long cutaneous horn on the center of forehead.

A 58-year-old gentleman, who is an agricultural laborer by occupation, presented to the outpatient department with a conical lesion over the center of his forehead (Figure 1) for the past three years.

The lesion was slowly increasing in size to attain the present size. The lesion was painless. He had no history of trauma and did not give any history of similar complaints. He was a chronic smoker for the past 15 years. He is a known case of well-controlled hypertension on regular medication. Owing to his daily outdoor working conditions, he had a prolonged history of sun exposure.

On examination, the lesion was 1.5 cm in length. It was non-tender and firm to hard in consistency. He had no other similar swellings over his body. Excision of the lesion along with the base was performed following which the histopathology examination showed features of benign seborrheic keratosis. There were no features of malignancy.

## Discussion

In the year 1588, cornu cutaneum was first documented in an elderly Welsh lady, Mrs. Margaret Gryffith [3]. They are relatively rare in Asian and African populations compared to Caucasians. This can be attributed to the protective action of melanin against ultraviolet radiation, which is believed to play a role in its etiology [4]. These cutaneous horns, although grossly similar, differ significantly from animal horns by the absence of an axial well-formed bone around which the hyperkeratotic epidermis and dermis are placed [6].

These lesions were previously associated with historic perplexity, and patients with these cutaneous horns were exhibited by money. Yu et al. had reported a totality of 643 patients with cutaneous horns over a period of 10 years with an annual frequency of 32 cases [7]. At around the same time, 48 patients were reported by Mencia-Gutierrez et al. [8].

The diagnosis is mostly clinical, and they are usually seen in association with several benign and malignant lesions at their bases. The horn being just an epidermal outgrowth of dead keratin is of little significance, but a striking aspect of this lesion is the underlying condition at its base which may be benign (seborrheic keratosis, keratotic acanthoma, viral wart, trichelemmoma, etc), premalignant (Bowen's disease, solar keratosis), or malignant (verrucous carcinoma [9], squamous cell carcinoma [10], basal cell carcinoma [11], epidermoid carcinoma, malignant melanoma) [3].

## Conclusions

Cutaneous horns are relatively common in Caucasian populations compared to African-Asian populations. Although most of these lesions are benign, there is a potential chance of these lesions harboring a malignant condition at their base that occurs in almost one-third of the cases.

Thus, excision of the lesion with its base along with histopathological examination forms the mainstay of the treatment. 
